# The Association Between Medication Adherence for Chronic Conditions and Digital Health Activity Tracking: Retrospective Analysis

**DOI:** 10.2196/11486

**Published:** 2019-03-20

**Authors:** Tom Quisel, Luca Foschini, Susan M Zbikowski, Jessie L Juusola

**Affiliations:** 1 Evidation Health San Mateo, CA United States; 2 inZights Consulting, LLC Seattle, WA United States; 3 Humana, Inc Louisville, KY United States

**Keywords:** activity tracker, health behavior, eHealth, mHealth, medication adherence

## Abstract

**Background:**

Chronic diseases have a widespread impact on health outcomes and costs in the United States. Heart disease and diabetes are among the biggest cost burdens on the health care system. Adherence to medication is associated with better health outcomes and lower total health care costs for individuals with these conditions, but the relationship between medication adherence and health activity behavior has not been explored extensively.

**Objective:**

The aim of this study was to examine the relationship between medication adherence and health behaviors among a large population of insured individuals with hypertension, diabetes, and dyslipidemia.

**Methods:**

We conducted a retrospective analysis of health status, behaviors, and medication adherence from medical and pharmacy claims and health behavior data. Adherence was measured in terms of proportion of days covered (PDC), calculated from pharmacy claims using both a fixed and variable denominator methodology. Individuals were considered adherent if their PDC was at least 0.80. We used step counts, sleep, weight, and food log data that were transmitted through devices that individuals linked. We computed metrics on the frequency of tracking and the extent to which individuals engaged in each tracking activity. Finally, we used logistic regression to model the relationship between adherent status and the activity-tracking metrics, including age and sex as fixed effects.

**Results:**

We identified 117,765 cases with diabetes, 317,340 with dyslipidemia, and 673,428 with hypertension between January 1, 2015 and June 1, 2016 in available data sources. Average fixed and variable PDC for all individuals ranged from 0.673 to 0.917 for diabetes, 0.756 to 0.921 for dyslipidemia, and 0.756 to 0.929 for hypertension. A subgroup of 8553 cases also had health behavior data (eg, activity-tracker data). On the basis of these data, individuals who tracked steps, sleep, weight, or diet were significantly more likely to be adherent to medication than those who did not track any activities in both the fixed methodology (odds ratio, OR 1.33, 95% CI 1.29-1.36) and variable methodology (OR 1.37, 95% CI 1.32-1.43), with age and sex as fixed effects. Furthermore, there was a positive association between frequency of activity tracking and medication adherence. In the logistic regression model, increasing the adjusted tracking ratio by 0.5 increased the fixed adherent status OR by a factor of 1.11 (95% CI 1.06-1.16). Finally, we found a positive association between number of steps and adherent status when controlling for age and sex.

**Conclusions:**

Adopters of digital health activity trackers tend to be more adherent to hypertension, diabetes, and dyslipidemia medications, and adherence increases with tracking frequency. This suggests that there may be value in examining new ways to further promote medication adherence through programs that incentivize health tracking and leveraging insights derived from connected devices to improve health outcomes.

## Introduction

### Background

Chronic diseases affect approximately half of all adults in the United States, and they are the leading cause of death and disability [1]. They also create a substantial cost burden; patients with chronic diseases accounted for 86% of all US health care spending in 2010 [2]. Of the biggest contributors, 2 are heart disease and stroke, estimated to cost US $315 billion in 2010, and diabetes, estimated to cost US $245 billion in 2012 [3,4]. Prescription medication is a key component of treatment for these diseases and their underlying risk factors, but adherence to medication has historically been low for patients with chronic diseases [5]. This is problematic as poor medication adherence can lead to poor health outcomes, which then increase health care utilization and costs [6].

The benefits of high medication adherence have been well established in diabetes, hypertension, and dyslipidemia, all of which are major risk factors for heart disease and stroke. One meta-analysis of studies in various disease areas, including diabetes and heart disease prevention, found that good medication adherence was associated with lower mortality, as compared with poor adherence [7]. Medical and pharmacy claims analyses have examined the relationship between medication adherence and health care utilization and costs. In 1 analysis across diabetes, dyslipidemia, and hypertension, the authors found that the annual total health care spending was significantly lower for adherent patients than nonadherent patients despite higher pharmacy costs in adherent patients [8]. The overall decrease in costs was driven by fewer hospitalizations and emergency department visits in the adherent population. Analyses of other claims data sources have led to similar conclusions [9-11].

### Objectives

Given the relationship between medication adherence and health care utilization and costs, efforts to increase medication adherence have been well studied. Patient behavior is often a key factor in individual medication adherence patterns, but research on the relationship between adherence and health activity behavior is limited [12]. Better understanding the link between behavior and medication adherence could facilitate the development of programs, tools, and approaches that improve adherence and thus lead to lower disease burden. With the recent proliferation of digital health trackers for activity, sleep, and diet, new data are available on these types of behaviors, which provide new opportunities to examine how health behaviors and lifestyles are linked to medication adherence. In 2013, an estimated 2% of the US population had used a wearable device. The use is growing quickly, with some estimates suggesting that over 20% of the population owned a wearable device in 2016 and annual sales projecting to increase to more than US $50 billion by 2018 [13,14]. We leveraged medical and pharmacy claims and other health behavior data from insured individuals to examine the relationship between health behavior and medication adherence. We sought to understand the connection between digital activity-tracking behavior and adherence for people with diabetes, dyslipidemia, and hypertension, and we sought to understand whether engaging with digital health trackers ties to changes in medication adherence. This can provide insight on the value of using data from connected devices to understand health behaviors such as medication adherence and improve health outcomes through health engagement strategies.

## Methods

### Study Sample

#### Claims Data

The analytic sample was derived from Humana medical and pharmacy claims and other health behavior data for an insured population. All data were deidentified and complied with requirements set forth by Humana’s Protected Health Information and Vendor Ethics committee. The study received institutional review board (IRB) exemption from Solutions IRB.

We identified cases with continuous health insurance coverage from January 1, 2015 to June 1, 2016 with at least 1 of 3 medical conditions: diabetes, dyslipidemia, or hypertension. Health conditions were established based on the International Classification of Diseases, Ninth Revision, Clinical Modification (ICD-9-CM) and ICD, Tenth Revision, CM (ICD-10-CM) codes, which are official systems of assigning codes to diagnoses and procedures associated with hospital utilization in the United States. Individuals were included if they had at least 1 outpatient visit or hospitalization with a specified ICD-9-CM or ICD-10-CM code, and they were included if there was a relevant diagnosis between January 1, 2005 to September 1, 2015 (Table 1). People with multiple conditions were included in each disease cohort.

The disease cohorts were further limited on the basis of pharmacy claims. We defined 2 periods for examining medication utilization: (1) the supply period and 2) the analysis period. The supply period (January 1, 2015-May 31, 2015) was the baseline time frame during which we utilized pharmacy claims to identify individuals who should be included in the analysis on the basis of prescription fills and to estimate the supply of medication in each patient’s possession at the start of the analysis period. Understanding the medication supply at the start of the analysis period is necessary for correctly computing medication adherence during that period. The analysis period (June 1, 2015-June 1, 2016) was the time frame in which we tracked prescription refills and adherence rates as well as activity-tracker use. To be included in the analysis, individuals must have had at least 1 pharmacy claim for an oral medication relevant to the disease cohort during the supply or analysis period (Table 1).

**Table 1:** Medical diagnosis codes used in condition cohort creation and classes of medication included in medication adherence analysis.

Condition	ICD-9-CM^a^ Codes	ICD-10-CM^b^ Codes	Pharmaceutical treatment classes
Diabetes	250.x	E08.x-E11.x	Alpha-glucosidase inhibitors; dipeptidyl peptidase‐4 inhibitors; glucagon-like peptide-1 receptor agonists; meglitinides; metformin/metformin combinations; sodium glucose cotransporter‐2 inhibitors; sulfonylureas; thiazolidinediones
Dyslipidemia	272.0, 272.2. 272.4	E78.0, E78.2, E78.4, and E78.5	Bile acid sequestrants; cholesterol absorption inhibitors; fibrates; lipid-regulating agents; nicotinic acid derivatives; statins
Hypertension	401.x-405.x	I10.x-I15.x	angiotensin-converting–enzyme inhibitors; Angiotensin II receptor blockers; beta blockers; calcium channel blockers; clonidine; diuretics; hydralazine; renin inhibitors

^a^ICD-9-CM: International Classification of Diseases, Ninth Revision, Clinical Modification.

^b^ICD-10-CM: International Classification of Diseases, Tenth Revision, Clinical Modification.

#### Activity-Tracking Data

Additional health behavior data were available for a subsample of participants who chose to use and link their activity-tracking device to track and earn points and status awards (eg, gold and silver) for participating in health and wellness activities. These points could be redeemed via a Web-based store for activity trackers, other fitness-related items (apparel, gear), and gift cards. We extracted activity-tracking data—including step counts, sleep duration, weight, and food logs. To focus on individuals who make a daily active choice of whether to track, we limited step counts in this analysis to those from tracker devices rather than step counts collected passively via smartphone.

### Study Variables and Analysis

The primary analysis examined the association between medication adherence, as measured by proportion of days covered (PDC), and activity-tracking metrics.

#### Medication Adherence

PDC is a common metric in retrospective medication adherence research, using pharmacy claims data to calculate the proportion of time that an individual had a prescribed medication on hand for a given condition [15]. We computed PDC using both a fixed and a variable denominator, as both methodologies are commonly used [8-11,16,17]. Fixed PDC generally serves as a lower bound for actual adherence, whereas variable PDC generally serves as an upper bound [18].

The fixed PDC methodology assumes that an individual should be taking medication during the entire analysis period [8,11]. Thus, we calculated the fixed PDC over a denominator of 1 year. The numerator is the number of days on which the individual had the medication on hand, including medication remaining from the supply period. In contrast, the variable PDC methodology assumes that the individual should be taking the medication only during the variable analysis period, defined as the time period between the first prescription fill and the end of the supply of the last refill [9]. Its numerator is the number of days on which the individual had the medication on hand during this period. Individuals must have had at least 2 prescription fills during the analysis period to prevent trivial variable PDC values of 1.0 from individuals who filled just 1 prescription during the analysis period [9]. For both the fixed and variable PDC methodology, PDC values ranged from 0.0 to 1.0.

We calculated a drug-class-level PDC over the analysis period for each therapeutic class of drugs used to treat the condition for which the individual filled prescriptions. We then calculated the condition-level PDC for each individual as an average of the per-individual drug-class-level PDCs for the fixed PDC methodology and as a weighted average of the per-individual drug-class-level PDCs for the variable PDC methodology. We used the length of the variable analysis period as the weight for each drug class’ variable PDC.

Finally, we used a threshold of condition-level PDC≥0.80 to define each individual as adherent or nonadherent for a particular condition. This is the threshold most commonly used in medication adherence research [5,6,8,9,19]. In the sensitivity analysis, we also considered a scenario where any individual with at least 1 drug-class-level PDC≥0.80 was classified as adherent.

#### Activity Tracking

We computed several activity metrics for each participant. First, we created a binary variable indicating if the individual had ever used an activity tracker during the analysis period (June 1, 2015-June 1, 2016). To understand how consistently individuals tracked their activities, we computed a tracking ratio for each individual: the ratio of days on which the individual logged at least 1 activity to the total number of days between the first activity logged and the most recent activity logged in the analysis period.

To correct for highly variable tracking ratios for individuals with little data, we created an adjusted tracking ratio. For each individual *m* we modelled the observed tracking ratio *r* = *k* / *n* (where *k* is the number of days tracked and *n* is the number of total days between *m*’s first and last day tracked) as a sample from a Binomial(*n*, *p*). To create a shrinkage estimator of *m*’s true tracking ratio using the Bayesian framework, we chose a Beta(α,β) distribution as our prior for *m*’s tracking ratio. We fixed α / (α+β) to *rmean*, the sample mean of tracking ratios across all individuals and fixed α+β-2 to 10. After performing a Bayesian update using *m*’s observed tracking ratio, Maximum A Posteriori estimation of *m*’s posterior tracking ratio was used to generate a point estimate of *m*’s true tracking ratio. With our choices of α and β, the point estimate reduced to (*nr* +10 *rmean*) / (*n* +10). We call this final result the adjusted tracking ratio.

Finally, to assess activity level, we computed steps taken per week during which a tracker was used. We divided the total number of steps taken by each individual in the measurement period by the number of weeks during which they tracked steps. We limited this metric to step count, which directly corresponds to the desired healthy behavior (ie, exercise). Sleep, weight, and food logging data were not used to assess activity level, as they do not have a direct linear tie to the desired healthy behavior (eg, sleep duration can be too short or too long and healthy weight is dependent on many other personal characteristics).

### Statistical Analysis

We performed all analyses using both the fixed and variable condition-level PDC adherent or nonadherent status, in turn, as the outcome variable. We considered a result significant if it achieved a *P* value of .05 or less using a 2-sided *t* test, or in the case of logistic regression, a 2-sided Wald test.

#### Association Between Adherence and Activity-Tracker User (Trackers vs Nontrackers)

We used logistic regression to assess the relationship between tracker versus nontracker status and fixed and variable adherent versus nonadherent status, including an interaction term to control for age and sex. We first built an overall model to measure the effect across all conditions and activities. Individuals with multiple conditions were accounted for under each condition. We then built 3 separate models, 1 for each condition, and then 4 additional models, 1 for each activity (steps, sleep, weight, and food logs), for a total of 7 models to investigate any differences in the tracker-adherence relationship across conditions or activity types.

#### Association Between Adherence and Activity-Tracking Metrics

We then used logistic regression to assess the relationship between fixed and variable individual adherent versus nonadherent status, frequency of tracking activities (as measured by adjusted tracking ratio), and activity level (as measured by steps per week tracked), including an interaction term to control for age and sex. We performed this analysis across individuals from all conditions, limited to those with at least 2 tracking events at least 10 days apart in the analysis period. Tracker data are significantly autocorrelated over the span of a few days, so the 10-day separation ensures at least 2 independent measurements per individual. We built a model for each of the 2 activity metrics independently as well as a combined model including both.

We performed all analysis using Python version 2.7.10 (Python Software Foundation), Spark version 1.3.1 (The Apache Software Foundation), Pandas version 0.15.2 (Python Software Foundation), and numpy 1.9.2 (NumPy Developers). We used significance tests from the stats module in scipy version 0.15.1 (Scipy Developers). We performed our logistic regressions using R version 3.2.0 (The R Foundation).

## Results

### Sample Characteristics

We identified 117,765 individuals with diabetes, 317,340 individuals with dyslipidemia, and 673,428 individuals with hypertension who were included in the fixed PDC analysis. Slightly fewer individuals qualified for the variable PDC analysis, as it required at least 2 pharmacy claims for at least 1 relevant medication—102,322 for diabetes, 286,640 for dyslipidemia, and 642,818 for hypertension. There was an overlap among the disease cohorts, ranging from 11.89% (37,719/317,340) of dyslipidemia individuals also having a diabetes diagnosis to 86.66% (102,050/117,765) of individuals with diabetes also having a hypertension diagnosis (Table 2). Coronary artery disease was also a common comorbidity, seen in approximately one-thirds of each cohort. On average, the population screened for diabetes was aged 70.5 years and 51.49% (60,641/117,765) females, for dyslipidemia was aged 70.8 years and 53.66% (170,289/317,340) females, and for hypertension was aged 70.8 years and 56.01% (377,183/673,428) females (Table 2). Individuals with tracker activity were younger with an average age of 52.3 years for diabetes, 55.1 years for dyslipidemia, and 52.5 years for hypertension (Table 3).

### Medication Adherence

Average PDC was similar across the conditions and was higher using the variable methodology than using the fixed methodology. Fixed methodology PDCs ranged from 0.673 for diabetes to 0.756 for dyslipidemia and hypertension, and variable methodology PDCs ranged from 0.917 for diabetes to 0.929 for hypertension (Table 4). The percent of individuals classified as adherent (PDC≥0.80) showed similar trends, ranging from 48.09% (56,630/117,765) for diabetes to 61.64% (195,606/317,340) for dyslipidemia for the fixed methodology and 85.38% (87,365/102,322) for diabetes to 89.07% (572,553/642,818) for hypertension for the variable methodology.

**Table 2:** Participant characteristics summarized by condition for all individuals.

Characteristic		Diabetes	Dyslipidemia	Hypertension
**Individual population, n**				
	Fixed PDC^a^ methodology	117,765	317,340	673,428
	Variable PDC methodology	102,322	286,640	642,818
Age (years), mean (SD)		70.5 (10.7)	70.8 (10.8)	70.8 (11.7)
**Sex, n (%)**				
	Female	60,641 (51.49)	170,289 (53.66)	377,183 (56.01)
	Male	57,124 (48.51)	147,051 (46.34)	296,245 (43.99)
**Comorbid conditions, n (%)**				
	Diabetes	117,765 (100)	37,719 (11.89)	102,050 (15.15)
	Dyslipidemia	37,719 (32.03)	317,340 (100)	235,863 (35.02)
	Hypertension	102,050 (86.66)	235,863 (74.33)	673,428 (100)
	Coronary artery disease	40,931 (34.76)	96,169 (30.30)	254,212 (37.75)
	Heart failure	16,745 (14.22)	35,936 (11.32)	81,362 (12.08)
	End stage renal disease	2534 (2.15)	5055 (1.59)	10,041 (1.49)
	Depression	12,085 (10.26)	29,655 (9.35)	78,035 (11.59)
**Digital tracking activity, n (%)**				
	None	116,974 (99.33)	313,741 (98.87)	666,956 (99.04)
	Steps	758 (0.64)	3498 (1.10)	6243 (0.93)
	Sleep	530 (0.45)	2460 (0.78)	4414 (0.66)
	Weight	179 (0.15)	736 (0.23)	1513 (0.22)
	Food logs	112 (0.10)	463 (0.15)	1050 (0.16)

^a^PDC: proportion of days covered.

**Table 3:** Participant characteristics summarized by condition for individuals with tracker data.

Characteristic		Diabetes	Dyslipidemia	Hypertension
**Individual population, n**				
	Fixed PDC^a^ methodology	791	3599	6472
	Variable PDC methodology	653	3196	6064
Age (years), mean (SD)		52.3 (10.2)	55.1 (9.6)	52.5 (10.5)
**Sex, n (%)**				
	Female	444 (56.1)	1813 (50.48)	3784 (58.47)
	Male	347 (43.8)	1786 (49.62)	2688 (41.53)

^a^PDC: proportion of days covered.

**Table 4:** Medication adherence by condition as measured by mean proportion of days covered and percent of individuals classified as adherent.

Metric		Diabetes	Dyslipidemia	Hypertension
**PDC^a^, mean (SD)**				
	Fixed PDC^a^ methodology	0.673 (0.320)	0.756 (0.291)	0.756 (0.256)
	Variable PDC methodology	0.917 (0.130)	0.921 (0.127)	0.929 (0.110)
**Adherent (PDC≥0.80), n (%)**				
	Fixed PDC methodology	56,630 (48.09)	195,606 (61.64)	373,515 (55.46)
	Variable PDC methodology	87,365 (85.38)	248,709 (86.77)	572,553 (89.07)

^a^PDC: proportion of days covered.

**Table 5:** Medication adherence metrics by age and sex. Results are significantly different between age groups and female versus male at *P*<.001 using a 2-sided *t* test.

Metric		Age		Sex	
		<50 years (n=43,545)	≥50 years (n=723,469)	Female (n=427,546)	Male (n=339,936)
**PDC^a^, mean (SD)**					
	Fixed PDC methodology	0.629 (0.321)	0.753 (0.271)	0.745 (0.275)	0.750 (0.275)
	Variable PDC methodology	0.882 (0.154)	0.928 (0.115)	0.925 (0.118)	0.927 (0.116)
**Adherent (PDC≥0.80), n (%)**					
	Fixed PDC methodology	17,785 (40.84)	414,009 (57.23)	238,638 (55.82)	194,503 (57.22)
	Variable PDC methodology	34,010 (78.10)	640,439 (88.52)	375,430 (87.81)	300,410 (88.37)

^a^PDC: proportion of days covered.

Medication adherence was significantly associated with age and sex. On the basis of age alone, there was a clear separation at 50 years between those who were more adherent and those who were less adherent. Adherence increased with age, with 40.84% (17,785/43,545) of individuals younger than 50 years being adherent across conditions as compared with 57.23% (414,009/723,469) of those aged 50 years and older, using fixed methodology (Table 5). Variable methodology showed a similar pattern, with 78.10% (34,010/43,545) of those younger than 50 years being adherent across conditions as compared with 88.52% (640,439/723,469) of those aged 50 years and older. Males were slightly but still significantly more adherent than females across conditions, with 57.22% (194,503/339,936) versus 55.82% (238,638/427,546) and 88.37% (300,410/339,936) versus 87.81% (375,430/427,546) being adherent under fixed and variable methodologies, respectively.

### Activity Tracking

We identified 8553 individuals who chose to link and share data from a digital health tracker for at least 1 activity across step counts, sleep, weight, and food logs, with step trackers being the most common. More than 75% of the individuals were using a Fitbit device to log steps and/or sleep, approximately 10% were logging steps via Garmin or Jawbone, and the remaining 15% were logging weight and food via Apple products (Watch and Health), MyFitnessPal, and RunKeeper, in decreasing order of prevalence. Tracker usage was more common in people younger than 50 years than in older populations, with 7.21% (3139/43,545) tracking at least 1 of the 4 activities compared with only 0.75% (5414/723,469) for individuals older than 50 years. Details on tracker usage by condition and type of activity can be seen in Table 2.

### Association Between Adherence and Activity-Tracker Use (Trackers vs Nontrackers)

**Figure 1:**
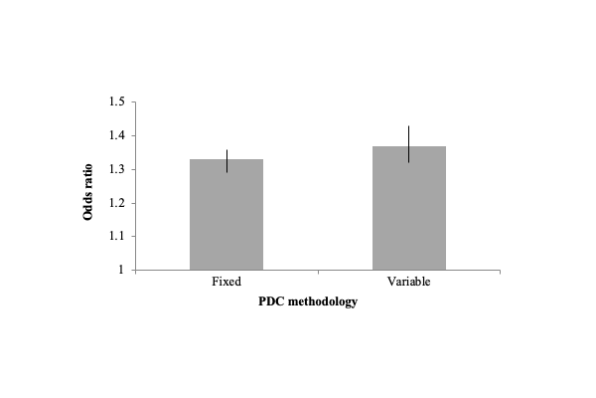
Odds ratios of percent of individuals who are adherent (proportion of days covered, PDC≥0.80) for individuals who use activity trackers versus nontrackers illustrate the association between activity-tracker use and medication adherence. Lines within the bars represent 95% CIs. Results are shown across conditions and activities tracked, while controlling for age and sex, and are significant at *P*<.001. PDC: proportion of days covered.

Across conditions, simply engaging in activity tracking was positively associated with medication adherence. When controlling for age and sex, people who tracked at least 1 of the 4 activities were significantly more adherent to medication than those who did not use any trackers, for both fixed adherent status (OR 1.33, 95% CI 1.29-1.36) and variable adherent status (OR 1.37, 95% CI 1.32-1.43; Figure 1). The results were similar when broken down by condition and specific activity tracked, with individuals who tracked a given activity more likely to be adherent than those who did not track the activity, for both fixed and variable methodologies and controlling for age and sex (Tables 6 and 7). The only exception was for diabetes in the fixed PDC model; the tracker-adherent relationship was not statistically significant (OR 1.12, 95% CI 0.96-1.30).

**Table 6:** The association between tracker use and medication adherence by condition in terms of the odds ratio of percent of individuals who are adherent (proportion of days covered≥0.80) for individuals who use activity trackers versus nontrackers. Odds ratios are significant at *P*<.001 unless otherwise noted.

Metric	Diabetes (n=791), OR^b^ (95% CI)	Dyslipidemia (n=3599), OR (95% CI)	Hypertension (n=6472), OR (95% CI)
Fixed PDC^a^ methodology	1.12 (0.96-1.30)^c^	1.23 (1.15-1.31)	1.38 (1.31-1.45)
Variable PDC methodology	1.31 (1.07-1.61)^d^	1.24 (1.13-1.37)	1.47 (1.36-1.59)

^a^PDC: proportion of days covered.

^b^OR: odds ratio.

^c^*P*=.14.

^d^*P*=.009.

**Table 7:** The association between tracker use and medication adherence by activity tracked in terms of the odds ratio of percent of individuals who are adherent (proportion of days covered≥0.80) for individuals who use activity trackers versus nontrackers. Odds ratios are significant at *P*<.001 unless otherwise noted.

Metric	Steps (n=10,499), OR^b^ (95% CI)	Sleep (n=7404), OR (95% CI)	Weight (n=2428), OR (95% CI)	Food logs (n=1625), OR (95% CI)
Fixed PDC^a^ methodology	1.34 (1.29-1.40)	1.35 (1.29-1.42)	1.21 (1.12-1.32)	1.20 (1.09-1.33)
Variable PDC methodology	1.37 (1.29-1.46)	1.38 (1.28-1.48)	1.37 (1.21-1.54)	1.26 (1.09-1.45)^c^

^a^PDC: proportion of days covered.

^b^OR: odds ratio.

^c^*P*=.001.

### Association Between Adherence and Activity-Tracking Metrics

Frequency of activity tracking and activity level were also tied to medication adherence. When controlling for age and sex, individuals who tracked activities more frequently or who were more active on the basis of step counts were significantly more likely to be adherent for both fixed adherent status and variable adherent status. In the logistic regression model, increasing the adjusted tracking ratio by 0.5 increased the fixed adherent status OR by a factor of 1.11 (95% CI 1.06-1.16) and the variable adherent status OR by a factor of 1.14 (95% CI 1.07-1.22). Increasing the steps-per-day-tracked by 2000 increased the fixed adherent status OR by a factor of 1.07 (95% CI 1.04-1.09) and the variable adherent status OR by a factor of 1.05 (95% CI 1.01-1.09). The combined logistic regression model, which included both adjusted tracking ratio and steps per week tracked to assess whether each predictor had an additive effect, gave similar results for the fixed methodology adherent status. However, in the combined variable adherent status model, activity level did not have a significant association with adherent status.

### Sensitivity Analysis

If any individual with at least 1 drug-class-level PDC≥0.80 was classified as adherent rather than basing the classification of adherent or nonadherent on whether the condition-level PDC was ≥0.80, engaging in activity tracking remained significantly and positively associated with medication adherence (fixed adherent status OR 1.21, 95% CI 1.18-1.25; variable adherent status OR 1.29, 95% CI 1.23-1.35). Just as in the base case, results were statistically significant when broken down by condition and specific activity tracked, except for diabetes in the fixed PDC model (OR 1.14, 95% CI 0.98-1.31). Frequency of tracking and activity level were also significantly tied to adherent status in the logistic regression model, except for the case of frequency of tracking for the variable adherent status.

## Discussion

### Principal Findings

Our analysis demonstrates that there is a significant relationship between medication adherence and activity tracking in individuals with chronic diseases, after controlling for age and sex. In particular, we found that people with diabetes, dyslipidemia, or hypertension who use activity trackers are more adherent to their medication than those who do not use activity trackers. In addition, medication adherence improves as consistency of tracker use and activity level increase. This analysis is an important first step in using additional available digital behavioral data to understand how health behavior ties to medication adherence.

These initial findings can be leveraged in a variety of clinically meaningful settings, such as targeting medication adherence initiatives. For example, considering that activity-tracking indices like the ones described require significantly shorter observation periods to be computed accurately for an individual as compared with PDC (days vs months), health plans or provider systems may use this information as a predictive score to selectively target new members for various medication adherence programs. Another use case is in enrollment for clinical trials of new chronic disease therapies. Trial recruitment can be targeted at patients who use activity trackers on a consistent basis, as these patients can be expected to have better medication adherence than nontracking patients of similar age and gender. This can minimize the sample size necessary for the trial. Targeting patients in this manner may be especially useful for enrolling younger patients, who tend to be less adherent yet significantly more likely to adopt activity trackers than older patients.

Our analysis also exposes many areas for further exploration into the relationship between patient behavior and medication adherence. We demonstrate a relationship between engaging in and consistency of activity tracking and medication adherence. One could suggest that individuals with tracker activity are being adherent to that intervention as they have a trait of behavior by which they are generally adherent to healthy activities and interventions. This is similar to the concept of the “healthy adherer”—the idea that healthier people are generally more adherent to medication than unhealthy people [6]. An interesting avenue of exploration is whether activity tracking is an independent indicator of adherence, in the sense that it has additional explanatory power in predicting medication adherence as compared with other healthy activities and interventions that show an association with medication adherence or adherence to other clinical care (eg, annual physical exams and glucose monitoring). Another future research avenue is to understand whether indices based on adherence to activity tracking are responsive to longitudinal changes in medication adherence at the individual level: does a sudden drop in activity tracking predict a time period of lower medication adherence? If this were to be the case, activity-tracking patterns could be used to responsively monitor medication adherence and deploy timelier interventions.

### Limitations

This study has several limitations. First, the findings are associative and do not demonstrate a causal link between adherence to activity tracking and medication adherence. Further research should be conducted to determine experimentally whether active manipulation of the activity-tracking behavior (eg, through incentives) leads to improved medication adherence. Second, only approximately 1% of the population in this study had data available from a linked digital health tracker, which limits the generalizability of study results. The study did not include traditional pedometers, and it did not include data from individuals who used digital devices but who did not link and share their data. Digital health tracker use is more common in younger populations than older adults; however, we expect applicability of these study results to grow over time as digital health tracker use continues to grow. This also may be enhanced as tracking capabilities, especially accelerometers for tracking steps, have become the standard for smartphones and thus are more accessible to wide audiences as adoption of new smartphones grows. However, it remains to be seen if the link uncovered between tracking and adherence will still be present for those who use built-in smartphone technology for tracking, as less effort is required to track activity in this scenario than purchasing or downloading a dedicated wearable or app.

This study is also subject to limitations common across claims-based analyses. Only insured individuals with pharmacy claims were included in the analysis; data from uninsured individuals or individuals who paid out of pocket for their medications were not captured. We used PDC to measure medication adherence, but it is an indirect measure of adherence that assumes filling a prescription equates to taking the medication. Individuals could fill but not consume prescriptions. This analysis also does not capture any medication discontinuations advised by a prescriber. Finally, we were only able to include interaction terms for age and sex in our analysis as other demographic factors such as income and education level were not available in the dataset.

### Conclusions

This study demonstrates that individuals who engage in activity tracking have significantly higher medication adherence than those who do not track their activities when controlling for age and sex across thousands of people with diabetes, hypertension, and dyslipidemia. The results were typically not dependent on a specific condition or activity tracked. The positive association with medication adherence extended to frequency of activity tracking as well as to physical activity level, as measured by step count. Given the well-established link between poor medication adherence and increased health care costs and utilization, as well as mortality, improving medication adherence in chronic conditions continues to be a high-value objective. This study is the first step in developing a better understanding of how to use digital health tools to understand and drive medication adherence and subsequently lower the cost of managing chronic diseases.

## Abbreviations

ICD-9-CM: International Classification of Diseases, Ninth Revision, Clinical Modification.
ICD-10-CM: International Classification of Diseases, Tenth Revision, Clinical Modification.
IRB: institutional review board
OR: odds ratio
PDC: proportion of days covered

## References

[1] Ward BW, Schiller JS, Goodman RA (2014). Multiple chronic conditions among US adults: a 2012 update. Prev Chronic Dis.

[2] Gerteis J, Izrael D, Deitz D, LeRoy L, Ricciardi R, Miller T, Basu J (2014). Agency for Healthcare Research and Quality.

[3] Go AS, Mozaffarian D, Roger VL, Benjamin EJ, Berry JD, Blaha MJ, Dai S, Ford ES, Fox CS, Franco S, Fullerton HJ, Gillespie C, Hailpern SM, Heit JA, Howard VJ, Huffman MD, Judd SE, Kissela BM, Kittner SJ, Lackland DT, Lichtman JH, Lisabeth LD, Mackey RH, Magid DJ, Marcus GM, Marelli A, Matchar DB, McGuire DK, Mohler ER, Moy CS, Mussolino ME, Neumar RW, Nichol G, Pandey DK, Paynter NP, Reeves MJ, Sorlie PD, Stein J, Towfighi A, Turan TN, Virani SS, Wong ND, Woo D, Turner MB, American Heart Association Statistics Committee and Stroke Statistics Subcommittee (2014). Heart disease and stroke statistics--2014 update: a report from the American Heart Association. Circulation.

[4] American Diabetes Association (2013). Economic costs of diabetes in the US in 2012. Diabetes Care.

[5] Osterberg L, Blaschke T (2005). Adherence to medication. N Engl J Med.

[6] Iuga AO, McGuire MJ (2014). Adherence and health care costs. Risk Manag Healthc Policy.

[7] Simpson SH, Eurich DT, Majumdar SR, Padwal RS, Tsuyuki RT, Varney J, Johnson JA (2006). A meta-analysis of the association between adherence to drug therapy and mortality. Br Med J.

[8] Roebuck MC, Liberman JN, Gemmill-Toyama M, Brennan TA (2011). Medication adherence leads to lower health care use and costs despite increased drug spending. Health Aff (Millwood).

[9] Pittman DG, Tao Z, Chen W, Stettin GD (2010). Antihypertensive medication adherence and subsequent healthcare utilization and costs. Am J Manag Care.

[10] Zhao Y, Zabriski S, Bertram C (2014). Associations between statin adherence level, health care costs, and utilization. J Manag Care Spec Pharm.

[11] Boye KS, Curtis SE, Lage MJ, Garcia-Perez L (2016). Associations between adherence and outcomes among older, type 2 diabetes patients: evidence from a Medicare Supplemental database. Patient Prefer Adherence.

[12] Brookhart MA, Patrick AR, Dormuth C, Avorn J, Shrank W, Cadarette SM, Solomon DH (2007). Adherence to lipid-lowering therapy and the use of preventive health services: an investigation of the healthy user effect. Am J Epidemiol.

[13] Brady CJ, Villanti AC, Pearson JL, Kirchner TR, Gupta OP, Shah CP (2014). Rapid grading of fundus photographs for diabetic retinopathy using crowdsourcing. J Med Internet Res.

[14] (2016). [PwC Sverige | Audit, Business Advice, Tax].

[15] Peterson AM, Nau DP, Cramer JA, Benner J, Gwadry-Sridhar F, Nichol M (2007). A checklist for medication compliance and persistence studies using retrospective databases. Value Health.

[16] Abughosh SM, Wang X, Serna O, Henges C, Masilamani S, Essien EJ, Chung N, Fleming M (2016). A pharmacist telephone intervention to identify adherence barriers and improve adherence among nonadherent patients with comorbid hypertension and diabetes in a medicare advantage plan. J Manag Care Spec Pharm.

[17] Slejko JF, Ho M, Anderson HD, Nair KV, Sullivan PW, Campbell JD (2014). Adherence to statins in primary prevention: yearly adherence changes and outcomes. J Manag Care Pharm.

[18] Kozma CM, Dickson M, Phillips AL, Meletiche DM (2013). Medication possession ratio: implications of using fixed and variable observation periods in assessing adherence with disease-modifying drugs in patients with multiple sclerosis. Patient Prefer Adherence.

[19] Halpern MT, Khan ZM, Schmier JK, Burnier M, Caro JJ, Cramer J, Daley WL, Gurwitz J, Hollenberg NK (2006). Recommendations for evaluating compliance and persistence with hypertension therapy using retrospective data. Hypertension.

