# Correction: The Association Between Medication Adherence for Chronic Conditions and Digital Health Activity Tracking: Retrospective Analysis

**DOI:** 10.2196/17375

**Published:** 2019-12-10

**Authors:** Tom Quisel, Luca Foschini, Susan M Zbikowski, Jessie L Juusola

**Affiliations:** 1 Evidation Health San Mateo, CA United States; 2 inZights Consulting, LLC Seattle, WA United States; 3 Humana, Inc Louisville, KY United States

In “The Association Between Medication Adherence for Chronic Conditions and Digital Health Activity Tracking: Retrospective Analysis” (J Med Internet Res 2019;21(3):e11486), a typesetting issue resulted in an error in Table 2 in the row “Comorbid conditions, n (%)”, subrow “Depression”.

The data was previously listed as follows:

- “Diabetes” column: 29,655 (9.35)
- “Dyslipidemia” column: 78,035 (11.59)
- “Hypertension” column: no data

However, the row was missing the value that should have been in the “Diabetes” column, so the two subsequent data points were misaligned into incorrect columns. The row now appears as follows:

- “Depression” column: 12,085 (10.26)
- “Dyslipidemia” column: 29,655 (9.35)
- “Hypertension” column: 78,035 (11.59)

The correction will appear in the online version of the paper on the JMIR website on December 10, 2019, together with the publication of this correction notice. Because this was made after submission to PubMed, PubMed Central, and other full-text repositories, the corrected article has also been resubmitted to those repositories.

